# Aqp 9 and Brain Tumour Stem Cells

**DOI:** 10.1100/2012/915176

**Published:** 2012-01-04

**Authors:** Guri Fossdal, Einar O. Vik-Mo, Cecilie Sandberg, Mercy Varghese, Mari Kaarbø, Emily Telmo, Iver A. Langmoen, Wayne Murrell

**Affiliations:** Vilhelm Magnus laboratory, Institute for Surgical Research, Norwegian National Hospital, Oslo University Hospital, 0450 Oslo, Norway

## Abstract

Several studies have implicated the aquaporins (aqp) 1, 4, and 9 in the pathogenesis of malignant brain tumours, suggesting that they contribute to motility, invasiveness, and oedema formation and facilitate metabolism in tumour cells under hypoxic conditions. We have studied the expression of aqp1, 4, and 9 in biopsies from glioblastomas, isolated tumour stem cells grown in a tumoursphere assay and analyzed the progenitor and differentiated cells from these cultures. We have compared these to the situation in normal rat brain, its stem cells, and differentiated cells derived thereof. In short, qPCR in tumour tissue showed presence of aqp1, 4, and 9. In the tumour progenitor population, aqp9 was markedly more highly expressed, whilst in tumour-derived differentiated cells, aqp4 was downregulated. However, immunostaining did not reveal increased protein expression of aqp9 in the tumourspheres containing progenitor cells; in contrast, its expression (both mRNA and protein) was high in differentiated cultures. We, therefore, propose that aquaporin 9 may have a central role in the tumorigenesis of glioblastoma.

## 1. Introduction

Since Peter Agres' discovery of the first water channel in 1992 [1], another 12 aquaporins have been described and linked to several diseases. Mainly, the aquaporin water channel family is divided into aquaporins and aquaglyceroporins as either exclusive water channels or water channels that also facilitate the transport of other solutes, reviewed by Borgnia et al. [2]. Gliomas are the most common primary brain tumours [3]. Due to their invasive and aggressive nature, this diagnosis bears a poor prognosis with a median survival of only one year from the time of diagnosis [4]. As for many other cancer types, an increasing body of evidence points to stem cells being culprits of tumour formation [5–9]. 

During recent years, several studies have shown that the aquaporins 1 and 4 [10–22] and the aquaglyceroporin 9 [17, 22–25] are overexpressed in tumours such as glioblastomas compared to normal brain tissue. This may, therefore, endow them with several of the characteristics of malignant brain tumours. For instance, aqp1 may play a role in their invasiveness [14] and in providing water influx into the expanding cellular protrusions. Also, tumour cells from astrocytomas and glioblastomas thrive under hypoxic conditions [26]. In this setting, aqp9 expression may account for their resistance to hypoxic and ischemic conditions, by facilitating clearance of lactate [27] and glycerol [28] resulting from hypoxia and cellular damage, respectively [29, 30]. It might, therefore, play a role in both the energy metabolism of normal brain tissue and provide increased tolerance for hypoxia under pathological conditions. Oedema formation is a major contributor to the morbidity and mortality associated with malignant brain tumours. Aqp4 is widespread throughout the brain parenchyma but especially enriched in glial cells neighbouring the ventricles, subarachnoid spaces, and blood vessels [31, 32]. This channel is polarized into the perivascular astrocytic end-feet processes together with the inward rectifying potassium channel Kir4.1 [33]. An aqp4-knockout model of water intoxication showed reduced oedema formation [34]. 

Using gene transfection to create knock-in and knockout experiments, these three aquaporins have been clearly implicated in cell motility [35–38]. Water permeability linked to F-actin stability in membrane leading edges and filopodia extension are suggested mechanisms. There is, therefore, an obvious implication for the migration of various cancer cell types.

In this study, we investigated the expression and localization of aquaporin 1, 4, and 9 in glioblastoma biopsies and in the tumour stem cells propagated as tumourspheres as well as differentiated cells isolated from these tumours. Rat tissue and cultured cells were used for comparison as indicators of the normal situation in the absence of human-derived tissue.

## 2. Materials and Methods

### 2.1. Tissue

Tumour tissue was obtained from brain tumour resections of glioblastomas performed at the Department of Neurosurgery at the University of Oslo, Faculty divisions of Rikshospitalet and Ullevål University Hospitals. The histopathological diagnosis and grade was established by neuropathologists according to the WHO classification [39]. Six patients were included in the study. Tissue harvesting was approved by the Norwegian National Committee for Medical Research Ethics. 

For all experiments with rodent neural stem cells, Wistar rats were used. The animals were of both sexes and from the age of four weeks and older. The animals were housed under conditions in accordance with the local Animal Research Committee. The animals were anaesthetized by inhalation of isoflurane as indicated by the manufacturer and rapidly decapitated.

### 2.2. Cell Culture

Rat tissue was harvested from the area of the lateral wall of the lateral ventricle in the forebrain/cerebrum. All brain tissue was transported in Leibovitz-15 medium (L-15) (BioWhittaker/Cambrex) on ice and processed as previously described [40]. The tissue was then mechanically dissociated with scissors and submitted to enzymatic treatment with either 0.5 mg/mL or 1x trypsin-EDTA (BioWhittaker/Cambrex) for human tumour tissue and cells, and 13.2 U/mL papain (Worthington) for rat tissue and cells. Finally, the suspension was filtered through a 70 *μ*m strainer (Falcon) and seeded at 400 000 viable cells/10 mL medium as indicated by the trypan blue exclusion method. The cells were cultivated as spheres in 25 mL flasks (NUNC) with DMEM/F12 (Invitrogen) in the presence of 20 ng/mL epidermal growth factor (EGF, R&D Inc., Minneapolis, Minn, USA), 10 ng/mL basic fibroblast growth factor (bFGF, R&D Inc.), 20 *μ*L/mL B-27 without vitamin A (Invitrogen), 10 mM Hepes, 10 *μ*L/mL Pen/Strep, and 2.5 *μ*g/mL heparin (LEO Pharma, Oslo, Norway). They were fed twice weekly. The spheres were cultured for five to seven days until reaching approximately 100 *μ*m in diameter, then enzymatically dissociated and reseeded. Dissociated cells from spheres were differentiated at 100 000 cells/dish on 40 mm glass-bottom dishes (Willco Wells BV, Amsterdam, The Netherlands) or 4-well slides (NUNC International) coated with 15 *μ*g/mL poly-L-ornithine (Sigma, St. Louis, Mo, USA). DMEM/F12 medium was augmented with fetal calf serum (3.75%; PAA Laboratories, Pasching, Austria) and 25 *μ*L/mL B-27 with vitamin A (Invitrogen), 1 *μ*g/mL laminin and 10 *μ*L/mL of Pen/Strep. All cultures were kept in an incubator at 5%-6% CO_2_, pH 7.2–7.4. 

### 2.3. Immunocytochemistry

#### 2.3.1. Preparation of Sections from Neurospheres and Cell Culture Dishes

For fixation of neurospheres, a few mL of medium was transferred to a 15 mL tube. The neurospheres were allowed to sink to the bottom of the tube by gravitation or easy centrifugation. Spheres were fixed in 4% paraformaldehyde, before being cryoprotected in 20% sucrose in PBS. Spheres were then frozen at −70°C, before being sectioned by cryotome at 10 *μ*m. Cell cultures were fixed with 4% paraformaldehyde for 30 min, followed by 2 × 5 min rinsing with PBS. The cells were incubated for one hour with a solution consisting of 1% Bovine serum albumin (BSA), 0.01% NaN_3_, 0.1% Triton-X100 diluted in PBS. Primary antibodies were incubated overnight at 4°C, rinsed in PBS, and incubated with secondary antibodies for 1 hour at 4°C in PBS containing 1% BSA and 0.3% Triton X-100 (Sigma-Aldrich). Control experiments were (1) no primary antibody, secondary antibody only to determine nonspecific binding of secondary antibody and (2) no antibody to determine the level of any autofluorescence. Analysis and image acquisition was done on an Olympus BV 61 FluoView confocal microscope (Olympus, Hamburg, Germany), using the FV10-ASW 1.7 software (Olympus). 

#### 2.3.2. Antibodies

The following primary antibodies were used: antiaquaporin 1 (rabbit polyclonal 1 : 500, Abcam ab15080), antiaquaporin 4 (rabbit affinity purified polyclonal 1 *μ*g/mL, Chemicon International), antiaquaporin 9 (rabbit polyclonal 5 *μ*g/mL, Alpha Diagnostic) (both latter aquaporin antibodies a kind gift from O. P. Ottersen), anti-HuNestin (mouse IgG1 1 : 1000, R&D Systems), anti-Sox2 (rabbit affinity purified polyclonal antibody 1 : 200, Chemicon International), anti-Doublecortin (DCX, goat affinity purified polyclonal 1 : 100, Santa Cruz Biotechnology, Inc.), anti-TuJ1 (rabbit affinity purified polyclonal antibody 1 : 1000, Sigma), and anti-GFAP (mouse monoclonal 1 : 1000, R&D Systems). Hoechst (nr. 33342 Sigma) was used for counterstaining of the nucleus. Secondary antibodies against the appropriate species used were labelled either with Alexa Fluor 488 (1 : 500, Molecular Probes), or Cy3 (1 : 1000, Jackson ImmunoResearch Laboratories). 

### 2.4. Reverse Transcription and Quantitative Polymerase Chain Reaction (q-PCR)

Tissue samples from normal SVZ and tumour were harvested in RNA*later* TissueProtect tubes (Qiagen). A portion of the cells from the sphere cultures was harvested by centrifugation and RNA isolated 24 h after passage. The remaining spheres were further cultivated and differentiated for 7 days before RNA isolation. Total RNA from tissue and cells was then isolated directly by using QIAzol (Qiagen, Germany) and the RNeasy Mini Kit (Qiagen, Germany) according to the manufacturer's protocol. The concentration of each RNA sample was determined by using the Nanodrop spectrophotometer and analyzed for quality visually with agarose gel electrophoresis.

Quantitative PCR was done using the standard curve method. Samples and standards (serial dilutions of total RNA) were reverse transcribed (RT) using 1 *μ*g total RNA and random hexamer priming (50 uL reaction volume, TaqMan Reverse Transcription Reagents from Applied Biosystems). Quantitative real-time PCR (qPCR) of each cDNA sample were run in triplicates using the TaqMan PCR Core Reagent Kit and the ABI Prism 7900 Sequence Detection System and software (Applied Biosystems, Foster City, Calif, USA) according to the manufacturer's instructions. The TaqMan aquaporin 1, 4, and 9 primers and detection probes were bought from Applied Biosystems. Primer sets for human aquaporins were AQP1: Hs00166067-m1, AQP4: Hs00242342-m1, and AQP9: Hs00175573-m1. Primer sets for rat aquaporins were AQP1: Rn01410034-m1, AQP4: Rn00563196-m1, and AQP9: Rn01530733-m1. For endogenous control reactions, 18S rRNA (TaqMan rRNA control reagents, Applied Biosystems) was used. The data acquired were analyzed with the Sequence Detector software (version 1.6.3, Applied Biosystems). Gene expression was presented relative to the levels of 18S ribosomal RNA. 

### 2.5. Preparation of Protein Extract and Western Analysis

Spheres were harvested, pelleted at 600 × g for 10 min at 4°C and washed in PBS. Adherent differentiated cells were washed in PBS, scraped, and pelleted as described above. The cells were homogenized using an eppendorf hand homogenizer in 0.3 M sucrose, 0.1 M NaPi, pH 7.4, 1 mM EDTA and protease inhibitors (Complete; Roche, Mannheim, Germany). The homogenates were centrifuged at 1,000 × g for 15 min, and the supernatants were collected. Sodium dodecyl sulphate (SDS) was added to the supernatants to a final concentration of 0.1%, incubated for 30 min at 4°C following centrifugation at 23,000 × g for 30 min at 4°C [41]. The supernatants from each sample containing cytosolic as well as membrane proteins were collected, and the amount of total protein was determined using the DC Protein Assay (Bio-Rad Laboratories, Hercules, Calif, USA). 10 *μ*g of whole protein extracts were loaded onto a 12% Nu-PAGE gel (Invitrogen) and subsequently blotted onto a 0.2 *μ*m PVDF membrane. The membrane was blocked with 5% skimmed milk in PBS/0.1% Tween 20 (PBST) and probed with rabbit *α*-human aquaporin 9 (1/1500, 85910, AbCam) or mouse *α*-human GAPDH (1/4000, Sigma) in TBST and 5% BSA. Secondary antibody was HRP-conjugated antirabbit IgG (1/5000 in 0.5% skimmed milk in TBST) or antimouse IgG (1/50 000 in 0.5% skimmed milk in TBST), respectively. The blots were developed using Super Signal West Dura ECL substrate (Pierce), detected by Kodak Molecular Imaging System (Kodak MI, version 5.0) and quantitated using the ImageQuant TL software (Amersham Biotech).

### 2.6. Statistics

The results were presented as a mean ± S.E.M. Differences were tested with unpaired two-sampled Student's *t*-test and considered significant when *P* < 0.05.

## 3. Results

We examined the mRNA gene expression of aqp1, 4, and 9 in the adult rat SVZ and primary tumours of human glioblastoma. Besides tissue, we also studied progenitor cells from free-floating neurospheres and cells of one week differentiation to reveal any changes associated with maturation. This analysis was accompanied by an assessment of expression at protein level using immunofluorescence and in the case of aqp9 with Western blot as well.

### 3.1. Aquaporin mRNA Expression in Rat Tissues and Cells (Table 1)

**Figure 1:**
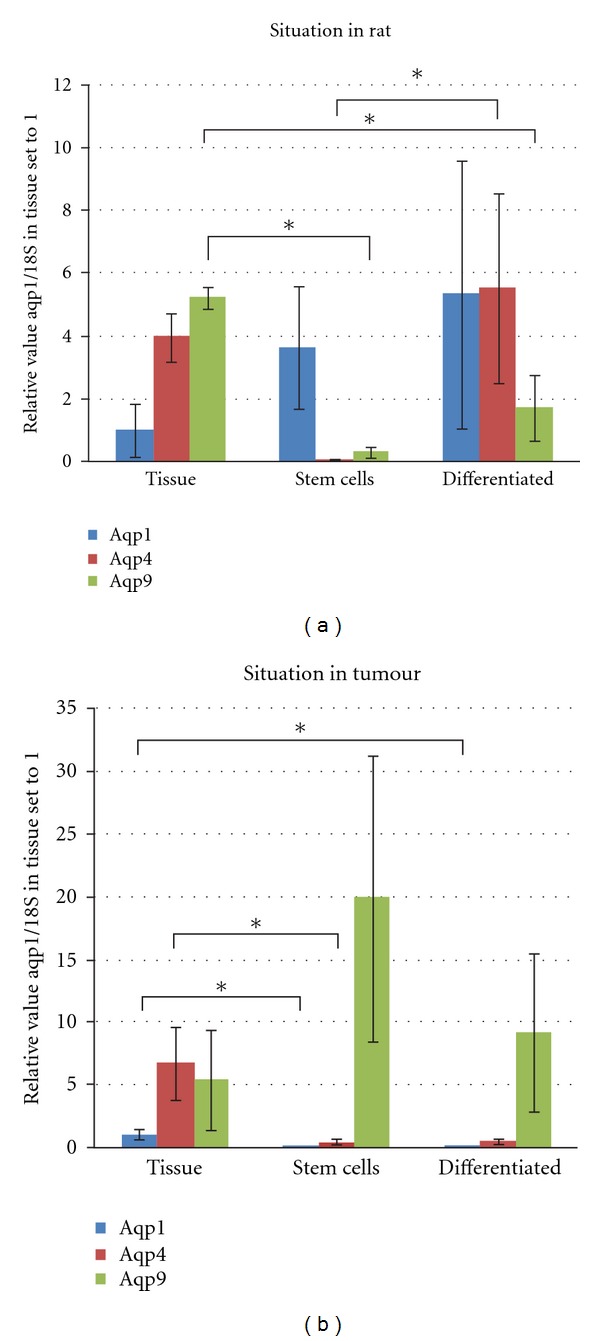
q-PCR analysis of mRNA expression of aquaporins 1, 4, and 9 in rat versus glioblastoma. (a) Levels of aquaporins in rat tissues. Progenitors mainly express aqp1. Differentiated cells expressed all three with aqp9 expressed the least. *values that differ statistically using unpaired two-sampled Student's *t-*test and considered significant when *P* < 0.05. (b) Levels of aquaporins in tumour tissues. In the progenitor and differentiated cell populations, aqp9 was conspicuously dominant compared to aqp1 and 4. *values that differ statistically using unpaired two-sampled Student's *t-*test and considered significant when *P* < 0.05.

In normal rat brain tissue, aqp9 was the highest expressed water channel, with a lesser amount of aqp4. The aquaporin/18S-ratio was estimated as 5.23 ± 0.32 and 3.98 ± 0.76, respectively (mean ± SEM, *n* = 4). Only small amounts of aqp1 were found (1.00 ± 0.83) in rat brain. Both neural progenitor and differentiated cells expressed strikingly higher levels of aqp1 than normal brain tissue. The highest level was in progenitors and was expressed at a much higher level than the other aquaporins. The individual results in this group were, however, highly scattered, and many of the progenitor and differentiated cell cultures had low expression of aquaporin (Figure 1). 

Also, we found significant upregulation of aqp4 in the differentiated cells compared to the rat neural progenitor population (Figure 1(a)). Aqp4 was very lowly expressed among the progenitor cells (0.04 ± 0.01), but differentiated cells displayed aqp4/18S ratio of 5.20 ± 3.01 (mean ± SEM, *n* = 8). In the case of aqp9 progenitor, and differentiated cells were calculated at 0.3 ± 0.2 and 1.70 ± 1.06, respectively (mean ± SEM, *n* = 4 (progenitor) and *n* = 7 (differentiated)).

### 3.2. Aqp9 Is Highly Expressed in the Progenitor Cells from Tumour

In tumour tissue, aqp4 expression was the most abundant (6.75 ± 2.86), but there was nearly as much aqp9 (5.37 ± 4.62) (mean ± SEM, *n* = 6, relative to aqp1 set to 1). When comparing the expression of aquaporin 9 by the tumour stem cells to that obtained directly from tissue, aqp9 was increased with respect to tissue, while aqp 1 and 4 were decreased (Figure 1(b)). Among the tumour stem cells, aqp9/18S was measured to 19.9 ± 6.35, whereas similar ratios for aqp1 and 4 were less, 0.02 ± 0.01 and 0.04 ± 0.18, respectively (mean ± SEM, *n* = 7, relative to aqp1 expression in tumour set to 1). 

### 3.3. Distinctive Behaviour of Aqp9

**Figure 2:**
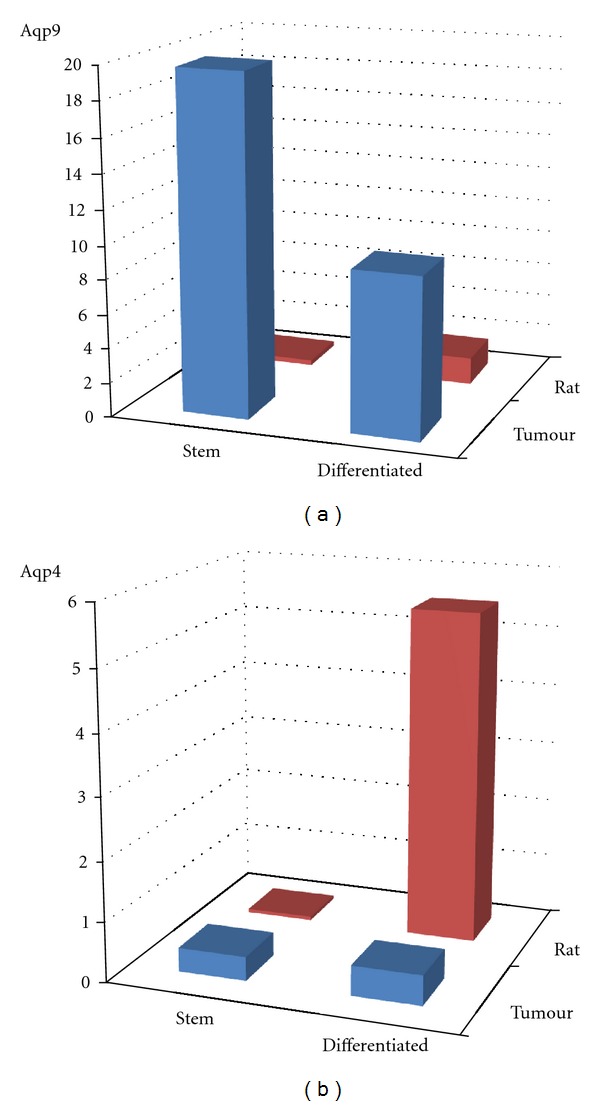
Distinctive behaviour of aqp9. Normal expression exhibited by rat progenitors and differentiated derivatives can be compared to that of tumour stem cells and derivatives. Whilst aqp4 expression does not markedly increase from tumour progenitor to differentiated cell, its expression increases markedly in rat differentiated cells. The situation for aqp9 is clearly different. Aqp9 is highly increased in both progenitor and differentiated derivatives when compared to normal rat tissue-derived cells.

Normal expression exhibited by rat progenitors and differentiated derivatives can be compared to that of tumour stem cells and derivatives (Figure 2). Whilst aqp4 expression does not markedly increase from tumour progenitor to differentiated cell, its expression does increase markedly in rat differentiated cells. The situation for aqp9 is clearly different. Aqp9 is highly increased in both tumour progenitor and differentiated derivatives when compared to normal rat tissue-derived cells.

### 3.4. Differentiated Tumour Cells Show Extensive Labeling for Aqp4 and 9

**Figure 3:**
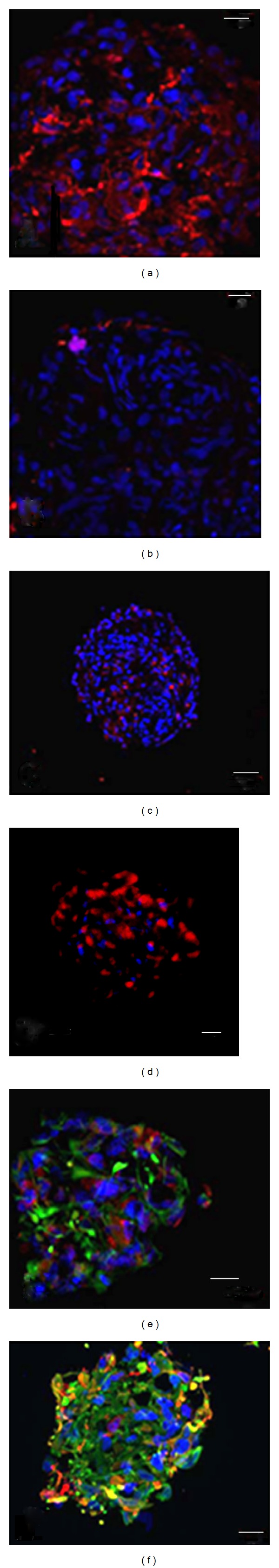
Expression of some proteins in tumourspheres. Tumourspheres from glioblastoma were mainly negative for the aquaporins 4 and 9 but were positive for immature and glial markers. (a, b) A few tumourspheres were weakly positive for aqp4 (a, red) but most were negative (b). (c) Few sphere cells were found to express aqp9. (d) Many sphere cells expressed aqp1. (e) A sphere showing the immature markers nestin (green) and Sox2 (red). (f) A sphere stained for nestin (green) and GFAP (red). Scale bars: (a, b, d, and e) 20 *μ*m; (c) 50 *μ*m.

The PCR experiments were supplemented with immunostaining of the aquaporins and other cellular markers to reveal their distribution among the different cell types. Tumourspheres were sectioned and stained and showed extensive labeling for the immature markers Nestin and Sox2. They were also positive for the glial marker GFAP which might reflect a more differentiated cell compartment in the sphere. However, few spheres were found positive for aqp4 and aqp9. These showed at most a rather week immunostaining (Figures 3(a), 3(b) and 3(c)). In contrast, many tumoursphere cells were positive for aqp1 (Figure 3(d)).

**Figure 4:**
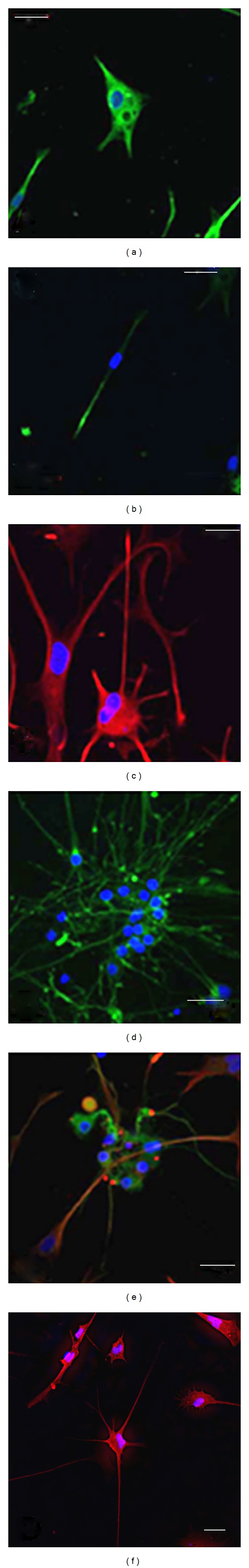
Differentiated cells. Tumour stem cells from glioblastoma differentiate into cells expressing aquaporins 1, 4, and 9 in addition to mature neuronal and glial markers. Yet, they retain their expression of immature markers. The cells displayed obviously aberrant morphology; note the multiple nuclei. (a, b): Differentiated cells of both glial and neuronal morphology stained positively for aqp4 (green). (c) Cells also exhibited extensive positive signal for aqp9 (red). (d) Nestin positive cells (green). (e) Cells positive for TuJ1 (red) and GFAP (green) Some cells display labelling with both antigens (green and red, together). (f) Most differentiated cells were positive for aqp1 (red). Scale bars: 20 *μ*m.

Tumour stem cells from glioblastoma differentiated into cells expressing mature neuronal and glial markers, TuJ1 and GFAP, respectively. Yet, they retained their expression of the immature marker nestin. The cells displayed aberrant morphology, with multiple atypical nuclei. Cells were immunopositive for aquaporins 1, 4, and 9. These aquaporins appeared widely distributed throughout cells—perhaps cytoplasms and membranes (Figures 4(a)–4(c) and 4(f)). Although there was no significant increase in the mRNA expression of aqp4 and 9 in differentiation of progenitor cells, the differentiated cells exhibited extensive labelling of aqp4 and 9 proteins compared to progenitor cells. Control experiments indicated no nonspecific staining of secondary antibodies or autofluorescence.

**Figure 5:**
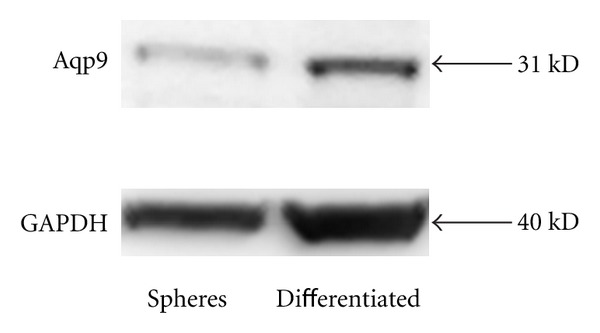
Western blot showing Aquaporin 9 protein expression in tumour progenitor cells (spheres) and cells differentiated from them. 100 *μ*g of protein was loaded in each lane.

A Western blot experiment (Figure 5) assessed protein levels of expression of aqp9 to be robust in both progenitors and differentiated cells suggesting that the immunolabeling experiments indicate differential accessibility, and, therefore, of location of antigen in sphere cells (Figure 4(c)).

## 4. Discussion

All three aquaporins were expressed in the rat brain tissue, and this was the situation too in human tumour tissue though aqp1 was lowest in both situations. In rat stem cells, aqp1 dominated, but all three were again expressed in differentiated cells. However, in the tumour stem cells and differentiated cells, only aqp9 dominated. This was, therefore, in stark contrast to the situation for normal brain (as evidenced in tissue and cells derived from rat). The immunofluorescence studies indicated that protein levels of aqp4 and 9 were low in tumour progenitors (tumourspheres) and highly expressed in the differentiated cells arising from them. As there seemed to be an inconsistency of expression of aqp9 at message level compared to immunolabeling of protein in the case of tumour progenitors, we carried out Western blot experiments of proteins extracted from progenitors and differentiated cells. These showed that although protein expression appeared lower in progenitors, it was still substantial if levels of the housekeeping gene GAPDH were compared. Thus, there would seem to be a difference in location within the cell or state of processing of aqp9 protein that inhibits detection by immunolabeling of intact (though permeablised) cells. 

In rat tissue, the low expression of aqp1 and substantially greater aqp4-expression were as expected, since aqp4 is found in abundance in astrocytes throughout the brain, while aqp1 appears only in the choroid plexus [42, 43]. However, the high expression level of aqp9 was somewhat more surprising but could be explained by expression in cells lining the ventricles [44]. Interestingly, none of the studies by Badaut et al. [29, 30] have shown labelling of the ependymal layer, but rather what seems to be a subependymal layer, and might represent the neurogenic subventricular zone (SVZ) [29]. This might correspond to our distinctive finding of a high expression of aqp9 in the stem cells from tumour, as further discussed below. Nevertheless, through mechanical isolation of the SVZ, there will inevitably be included both ependymal and subependymal material, which is very thin and tightly adherent to the underlying SVZ [45]. 

Due to the difference in species and different PCR probes for aquaporins in rat and human, human tumour can, of course, only be quantitatively compared to normal human tissue. And although we have not performed PCR analysis of normal adult human brain, we would have expected to find, but did not compared to rat, an increase for all three channels, as tumour tissue has exhibited profoundly increased immunohistochemical labeling in comparisons between normal and tumour brain tissue. The upregulation has been supported at protein level by immunoblot [10, 13–17, 26, 46].

Both aqp4 and 9 expression in tumour tissue appears robust. A common MAP kinase pathway is found to be involved in the regulation of both aqp4 and 9 channels under hypoxic conditions [47]. Another study has found evidence for regulation through protein kinase C for both aqp4 and 9 [48]. This may imply that these two aquaporins at least share some pathways of regulation in common. 

Although we have not performed immunocytochemistry on differentiated cells from normal human brain, the differentiated tumour cells clearly differed from their normal counterparts found in brain tissue according to published data. Normal astrocytes display a polarized distribution of the aqp4 in brain tissue [31], as this is found only on perivascular end feet, together with the inward rectifying K+-channel 4.1 [25, 49, 50]. Together, they enable directed water flow. It has been shown in several studies that in various pathological conditions like injury [51], ischemia [52], subarachnoid haemorrhage, SAH [47] and brain tumour [53–55], polarization is lost, rendering aqp 4 highly distributed throughout the entire cell. This is similar to the labelling distribution we found in differentiated tumour stem cells but as in vitro conditions obviously differ greatly from those of in vivo, further experimental evaluation is required. 

One possible explanation for this might be downregulation or dysfunction of the anchoring proteins for the aquaporins. In a study by Warth et al. [23], polarization of aqp4 was totally dependent on its binding through the agrin protein for linking to the dystrophin complex, a cell-surface receptor that links the cytoskeleton to the extracellular matrix [56]. Agrin is, on the other hand, an extracellular heparin sulphate proteoglycan and is previously shown to be downregulated in glioblastoma [57]. In another study, Warth et al. [17] found that this phenomenon is a function of malignancy, as high-grade tumours showed a highly exaggerated and randomly distributed aquaporin 4 channel towards the entire cell membrane. Agrin is also linked to the vascular endothelial membrane and upon downregulation causes several tight junction proteins to disappear, disrupting the blood brain barrier [58]. The agrin protein might, therefore, be central for the development of brain oedema, acting in concert with other proteins like aqp4 and tight junction proteins.

**Does Aqp9 Exhibit a Special Role for the Tumorigenicity of Tumour Stem Cells?**

When comparing the expression of aquaporin 9 among the progenitor cells to that obtained directly from tissue, aqp9 is increased with respect to tissue, while aqp1 and 4 are decreased. Aqp9 is many times more highly expressed by tumour stem cells and their differentiated progeny than is the case in normal rat-derived cells. Furthermore, the high level of aqp9 transcription detected in the tumour-derived progenitor cells not evident yet at protein level—we show that it is lowly expressed using immunofluorescent staining and moderately expressed as assessed for cell lysates using Western blot—is a prelude for high expression of both mRNA and protein of this gene in the “differentiated” cells arising from them. This implies a central role for aqp9 in the progenitor cell population in glioblastoma. Aquaporin 9 is also found in the cytoplasms and cell processes of astrocytes [53] but does not display the universal preference for pericapillary astrocytic membranes as is the case for aqp4 [59].

The marked upregulation of aqp9 mRNA in tumour stem cells (grown in culture as neurospheres) followed by high expression at both message and protein level in “differentiated” cultures growing adherently in vitro implies a role for this water channel in the life process of the tumour. Perhaps ability of migrating tumour stem cells to colonise surrounding normal tissue is somehow advantaged by the expression of aqp9 water channel as some of the cells acquire a more “differentiated” phenotype.

If these proteins play a role in the tumorigenicity of tumour stem cells, the aquaporins, aqp9 in particular, may be candidates for targeted immunotherapy, as at least their extracellular epitopes should be accessible for antibodies.

## Figures and Tables

**Table 1:** Relative mRNA expression of Aquaporins 1, 4, and 9 in normal rat brain and human brain tumour-derived tissue, stem cells, and differentiated cells. Values are based on setting the ratio of Aqp1/18S in tissue to 1.

	Rat		Tumour	
	Mean value	SEM	Mean value	SEM
Aqp1 Tissue	0.999999	0.824987	1	0.348343
Aqp4 Tissue	3.979995	0.763569	6.75893	2.862152
Aqp9 Tissue	5.23405	0.320761	5.375927	4.025013

Aqp1 Spheres	3.641413	1.945498	0.016193	0.007146
Aqp4 Spheres	0.039853	0.005692	0.424486	0.179481
Aqp9 Spheres	0.306192	0.162423	19.903079	11.36836

Aqp1 Differentiated	5.330058	4.272465	0.006569	0.002806
Aqp4 Differentiated	5.527011	3.008557	0.493593	0.190121
Aqp9 Differentiated	1.706198	1.062908	9.1752	6.359034
